# Inappropriate Emergency Department Visits: Insights on Incidence, Associated, and Predictive Factors From 5,429 Visits

**DOI:** 10.7759/cureus.65091

**Published:** 2024-07-22

**Authors:** Abdulaziz M Alghamdi, Mohamed K Alqazenli, Mohammed I Alzahrani, Nawaf A Bin Khamis, Ghadeer A Al Yusuf, Tajah M Alaithan, Hind H Alshobaki, Muhnnad A AlGhamdi, Mouath H Asiri, Sawsan T Hanafi

**Affiliations:** 1 College of Medicine, King Saud Bin Abdulaziz University for Health Sciences, Jeddah, SAU; 2 Research Office, King Abdullah International Medical Research Center, Jeddah, SAU; 3 College of Medicine, Medical University of Warsaw, Warsaw, POL; 4 College of Medicine, AlMaarefa University, Riyadh, SAU; 5 College of Medicine, Ibn Sina National College for Medical Studies, Jeddah, SAU; 6 Emergency Medicine, Ministry of National Guard Hospital, Jeddah, SAU

**Keywords:** non-urgent, urgent medical cases, healthcare quality, customized patient care, inappropriate visit, emergency department, emergency medicine

## Abstract

Introduction: Inappropriate visits (IVs) and overcrowded emergency departments (EDs) can result in many complications for patients and medical staff. This study aimed to assess the incidence, associated factors, and predictive factors of IVs to ED.

Methods: This retrospective cohort single-center study was conducted in the ED of King Abdulaziz Medical City, Jeddah, Saudi Arabia. All ED visits in February 2023 were included. They were considered appropriate if the patient required investigation tests, underwent a procedure, was admitted to an inpatient ward, was admitted to the short-stay unit, was referred for follow-up at a specialist outpatient clinic after discharge from the ED, or was referred to the ED of another hospital. Failure to have at least one of these factors led to the visit being considered inappropriate.

Results: A total of 5,429 visits were included. The incidence rate of IVs was 1128 (20.7%). Of the visits, 1,028 (18.9%) were attended by patients aged <10 years, and 2,825 (52.0%) by female patients. The most reported complaints were pulmonological in 1,029 visits (18.9%). Patients with appropriate visits (AVs) had significantly higher median BMI scores than those with IVs (25.9 (20 - 3) vs. 23.7 (16.36 - 29), *P *= <0.0001). Visits with pulmonological (447 (39.6%) vs. 582 (13.5%)) and otorhinolaryngologic (54 (4.7%) vs. 94 (2.1%)) complaints were significantly more likely to be inappropriate (*P *= <0.0001). In multiple logistic regression, being a male (OR: 1.3, CI: 1.1 - 1.5, *P *= <0.0001), being non-Saudi (OR: 2.7, CI: 2.0 - 3.6, *P *= <0.0001), and visiting on the weekend (OR: 1.1, CI: 1.0 - 1.3, *P *= 0.0366) were significantly predictive of the visits being inappropriate.

Conclusions: Our findings revealed a high incidence of IVs in the ED, with several factors predictive of IVs. Highlighting these factors can help minimize the incidence of IVs and, therefore, improve the quality of healthcare delivered to patients in need and their clinical outcomes.

## Introduction

The Emergency Department (ED) is an integral service of the healthcare system that provides an immediate 24-hour point of access to care for patients with urgent medical conditions [1,2]. The department relies on qualified and trained specialized medical teams to respond promptly, rapidly assess, accurately diagnose, treat emergencies, and cooperate with other hospital departments. Inappropriate visits (IVs) and overcrowded EDs are major problems in many countries worldwide. IVs to EDs are defined as self-referred patients or inappropriate referrals by primary care physicians with non-urgent conditions that are better managed by other services [3]. IVs can result in many issues for patients and medical staff, including deterioration of the patient’s health and psychological condition as a result of the long waiting time, loss of control over critical cases, frustration among the medical staff, decrease in the doctor’s productivity, and increased medical errors [3-5].

Many international multicenter studies with large numbers of visits have been conducted to assess the incidence of IVs and/or their related factors, and their results have helped further manage such groups of patients [1-6]. However, in Saudi Arabia, only a few studies have partially addressed this topic [7,8]. Identifying the incidence and related factors of IVs will help us in many aspects to deliver the best healthcare possible to patients in need who are considered to have appropriate visits (AVs) to the ED and guide others to the right places where they can also receive medical treatment. This study aimed to assess the incidence, associated factors, and predictive factors of IVs in the ED at the National Guard Hospital, Makkah region, Saudi Arabia.

## Materials and methods

This retrospective chart review cohort study was conducted at King Abdulaziz Medical City, Jeddah, Saudi Arabia, in February 2023. Ethical approval was obtained from King Abdullah International Medical Research Center, Jeddah, Saudi Arabia, under protocol number NRJ23J/080/03. This study aimed to assess the incidence, associated factors, and predictive factors of IVs in the ED. All patients of any age and sex who attended the ED in February 2023 and were seen by physicians with complete documentation in the system were included in this study. 

Data were collected using the hospital system. The data collection sheet included data on age, sex, nationality, Body Mass Index (BMI), day of attendance, time of attendance, source of referral, presenting complaint, and whether the visit was appropriate. The day of attendance was marked as a weekday or weekend. The time of attendance at the ED was marked as morning (08:00 a.m.-04:00 p.m.), evening (04:00 p.m.-12:00 a.m.), or nighttime (12:00 a.m.-08:00 a.m.). The presenting complaints were categorized based on the symptoms of the main body system, the patients were presented with, and their final diagnoses. 

Finally, the visits were categorized as AV or IV based on the pre-specified criteria published by Oh et al. in 2020 [9]. The criteria have been used by the ED at Changi General Hospital, Singapore, since early 2014 to classify the appropriateness of general practitioner referrals. Also, this retrospective classification of ED visits based on the same criteria was found to be consistent with the opinions of senior ED physicians in the same hospital after they had reviewed the medical records of the sampled attendances [9]. Their criteria stated that any patient who required an investigation, or required a procedure, was admitted to an inpatient ward, was referred for follow-up at a specialized outpatient clinic after discharge, or was referred to the ED of another hospital was considered to have an AV. Any patient who failed to have at least one of these elements was considered to have an IV. 

The collected data were analyzed using John's Macintosh Project (JMP) statistical software version 15.2.0 (SAS Institute, Cary, NC, a subsidiary of the SAS Institute). Qualitative variables were presented as frequencies and percentages. Quantitative variables were presented as medians and interquartile ranges. The association between two categorical variables was assessed using the chi-squared test. A t-test was used to assess the association between numerical and categorical variables. Multiple logistic regression analysis of the significantly associated factors was used to assess the predictive factors for IV in the ED. Statistical significance was set at *P* < 0.05, and the corresponding 95% confidence interval (CI) was set.

## Results

**Figure 1:**
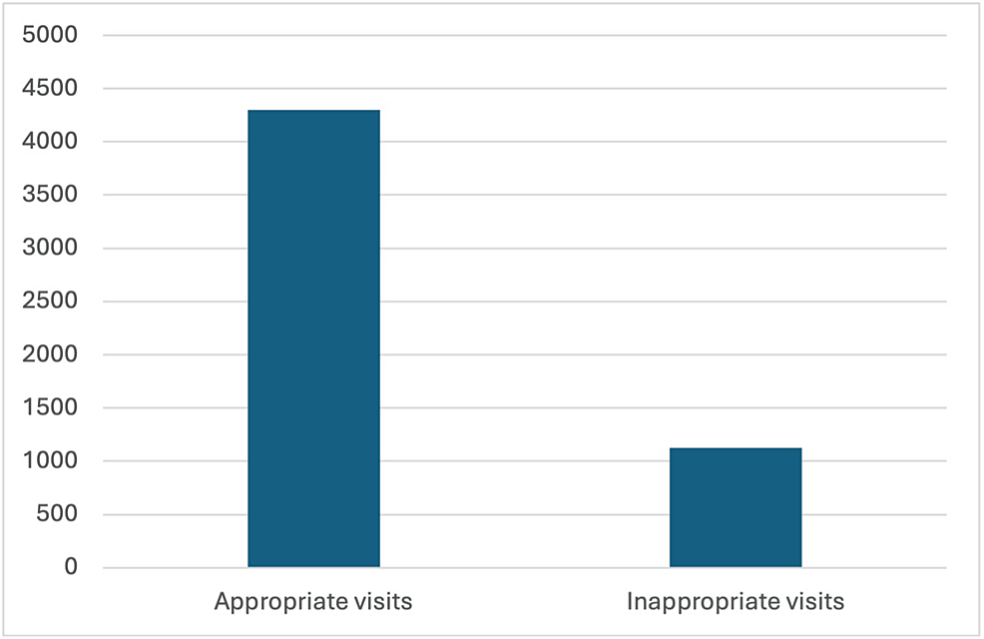
Distribution of numbers of appropriate and inappropriate visits. Data has been presented as numbers.

In total, 5,429 ED visits were included in this study. A total of 1,128 visits were found to have IVs with an incidence of 20.7% (Figure 1). Among the total number of visits, patients aged <10 years had the highest number with 1,028 visits (18.9%) (Table 1). Female patients constituted 2,825 visits (52.0%). For the source of referral, 5,332 visits (98.2%) were self-referrals (Table 1).

**Table 1:** The baseline characteristics of emergency department visits. IQR: Interquartile range. The data has been represented as numbers (percentages) and medians (interquartile ranges). A *P*-value <0.05 was considered significant.

Variable	Total visits (n = 5429)	Appropriate visits (n = 4301)	Inappropriate visits (n = 1128)	*P*-value
Age in years, n (%)	-	-	-	<0.0001
<10	1028 (18.9)	682 (15.8)	346 (30.6)	-
10-19	392 (7.2)	323 (7.5)	69 (6.1)	-
20-29	768 (14.1)	591 (13.7)	177 (15.6)	-
30-39	956 (17.6)	686 (15.9)	270 (23.9)	-
40-49	507 (9.3)	394 (9.1)	113 (10.0)	-
50-59	549 (10.1)	481 (11.1)	68 (6.0)	-
60-69	578 (10.6)	528 (12.2)	50 (4.4)	-
≥ 70	651 (11.9)	616 (14.3)	35 (3.1)	-
Gender, n (%)	-	-	-	<0.0005
Male	2604 (47.9)	2011 (46.7)	593 (52.5)	-
Female	2825 (52.0)	2290 (53.2)	535 (47.4)	-
Nationality, n (%)	-	-	-	<0.0001
Saudi	5220 (96.1)	4175 (97.0)	1045 (92.6)	-
Non-Saudi	209 (3.8)	126 (2.9)	83 (7.3)	-
BMI, median (IQR)	25.28 (19-30.4)	25.95 (20-3)	23.71 (16.36-2)	<0.0001
Day of attendance, n (%)	-	-	-	<0.0140
Weekdays	3950 (72.7)	3162 (73.5)	788 (69.8)	-
Weekend	1479 (27.2)	1139 (26.4)	340 (30.1)	-
Time of attendance, n (%)	-	-	-	0.4792
Morning time	1745 (32.1)	1395 (32.4)	350 (31.0)	-
Evening time	2234 (41.1)	1772 (41.2)	462 (40.9)	-
Nighttime	1450 (26.7)	1134 (26.3)	316 (28.0)	-
Source of referral, n (%)	-	-	-	0.0011
Self-referral	5332 (98.2)	4209 (97.8)	1123 (99.5)	-
Primary healthcare	58 (1.0)	56 (1.3)	2 (0.1)	-
Ambulance	18 (0.3)	18 (0.4)	0	-
Other healthcare institutions	21 (0.3)	18 (0.4)	3 (0.2)	-

**Figure 2:**
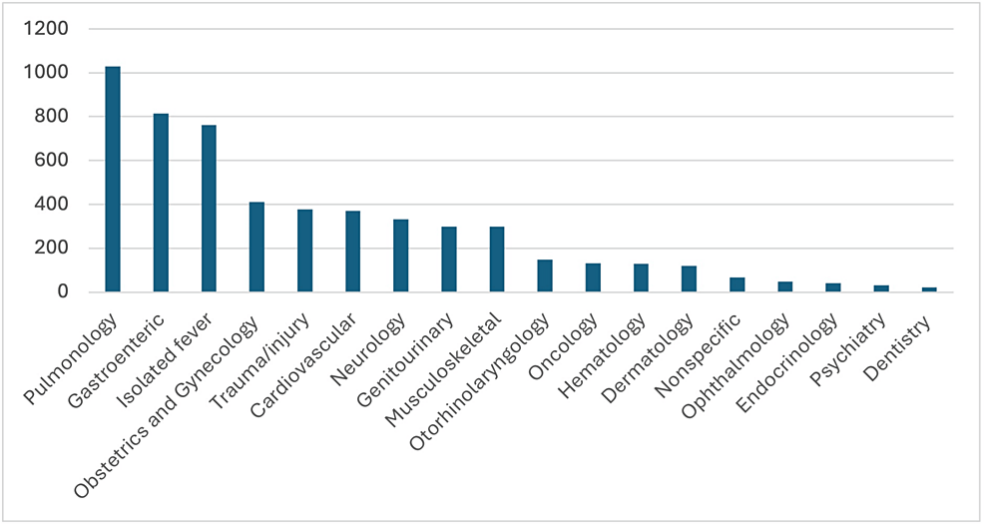
Distribution of numbers of different presenting complaints based on the body systems. Data has been presented as numbers.

The most reported complaints by ED visits were pulmonological in 1,029 (18.9%), gastroenteric in 815 (15.0%), and isolated fever in 761 (14.0%) (Table 2) (Figure 2). 

**Table 2:** The presenting complaints of emergency department visits. The data has been represented as numbers (percentages). A *P*-value <0.05 was considered significant.

Variable	Total visits (n = 5429)	Appropriate visits (n = 4301)	Inappropriate visits (n = 1128)	*P*-value
Presenting complaint, n (%)	-	-	-	<0.0001
Trauma/injury	377 (6.9)	350 (8.1)	27 (2.3)	-
Isolated fever	761 (14.0)	571 (13.2)	190 (16.8)	-
Gastroenterology	815 (15.0)	680 (15.8)	135 (11.9)	-
Cardiovascular	371 (6.8)	359 (8.3)	12 (1.0)	-
Pulmonology	1029 (18.9)	582 (13.5)	447 (39.6)	-
Neurology	332 (6.1)	293 (6.8)	39 (3.4)	-
Musculoskeletal	298 (5.4)	219 (5.0)	79 (7.0)	-
Hematology	129 (2.3)	123 (2.8)	6 (0.5)	-
Oncology	131 (2.4)	124 (2.8)	7 (0.6)	-
Endocrinology	41 (0.7)	37 (0.8)	4 (0.3)	-
Obstetrics and gynecology	411 (7.5)	381 (8.8)	30 (2.6)	-
Genitourinary	298 (5.4)	283 (6.5)	15 (1.3)	-
Dermatology	120 (2.2)	90 (2.0)	30 (2.6)	-
Otorhinolaryngology	148 (2.7)	94 (2.1)	54 (4.7)	-
Ophthalmology	47 (0.8)	34 (0.7)	13 (1.1)	-
Psychiatry	31 (0.5)	27 (0.6)	4 (0.3)	-
Dentistry	22 (0.4)	12 (0.2)	10 (0.8)	-
Nonspecific	68 (1.2)	42 (0.9)	26 (2.3)	-

The visits made by patients aged <10 years (346 (30.6%) vs. 682 (15.8%)) and 30-39 (270 (23.9%) vs. 686 (15.9%)) were significantly more likely to be inappropriate (*P* = <0.0001). Visits by female patients were significantly less likely to be inappropriate (2290 (53.2%) vs. 535 (47.4%), *P* <0.0005). Non-Saudi patients’ visits were significantly more likely to be inappropriate (83 (7.3%) vs. 126 (2.9%), *P* = <0.0001). Patients with AVs had significantly higher median BMI scores than those with IVs (25.9 (20 - 31) vs. 23.7 (16.3 - 29), *P* = <0.0001). Visits on the weekend were more likely to be inappropriate (340 (30.1%) vs. 1139 (26.4%), *P* = <0.0140). The remaining findings are presented in Table 1. 

Visits with pulmonological (447 (39.6%) vs. 582 (13.5%)) and otorhinolaryngological (54 (4.7%) vs. 94 (2.1%)) complaints were significantly more likely to be inappropriate (*P* = <0.0001). The remaining findings are presented in Table 2.

Multiple logistic regression analysis of the significantly associated variables was conducted to assess the predictive factors for IVs in the ED. Being a male (OR: 1.3, CI: 1.1 - 1.5, *P* = <0.0001), being non-Saudi (OR: 2.7, CI: 2.0 - 3.6, *P* = <0.0001), and visiting on the weekend (OR: 1.1, CI: 1.0 - 1.3, *P* = 0.0366) were significantly predictive of the visits being inappropriate. 

On the other hand, interestingly, taking the age of <10 as a reference, all age categories were significantly negative predictors of IVs, with the age category ≥ 70 years old (OR: 0.1, CI: 0.0 - 0.1, *P* = 0.0001) having the lowest odds ratio and the age category 30-39 years old (OR: 0.7, CI: 0.6 - 0.9, *P* = 0.0294) having the highest odds ratio. In addition, visits made by patients referred from primary healthcare (PHC) were significantly negative predictors of IVs (OR: 0.1, CI: 0.0 - 0.6, *P* = 0.0116). The rest of the results are in Table 3.

**Table 3:** Multiple logistic regression analysis of predictive factors of inappropriate emergency department visits. NA: Not applicable, BMI: Body Mass Index. The data has been represented as odds ratios, lower confidence intervals, and upper confidence intervals. A *P*-value <0.05 was considered significant. In each variable that has been included in the multiple logistic regression analysis, the group with the highest frequency has been used and labeled as a reference of comparison for the other groups within the same variable. Any group that had a very low frequency to be included in the multiple logistic regression analysis has been labeled as not applicable.

Variable	Odds ratio	Lower confidence interval	Upper confidence interval	*P*-value
Age	-	-	-	-
<10	Reference	Reference	Reference	Reference
10-19	0.4	0.2	0.5	<0.0001
20-29	0.6	0.4	0.8	<0.0001
30-39	0.7	0.6	0.9	0.0294
40-49	0.5	0.4	0.7	0.0001
50-59	0.3	0.2	0.4	<0.0001
60-69	0.1	0.1	0.2	<0.0001
≥ 70	0.1	0.0	0.1	<0.0001
Gender	-	-	-	-
Male	1.3	1.1	1.5	<0.0001
Female	Reference	Reference	Reference	Reference
BMI	0.9	0.9	1.0	0.4327
Nationality	-	-	-	-
Saudi	Reference	Reference	Reference	Reference
Non-Saudi	2.7	2.0	3.6	<0.0001
Day of attendance	-	-	-	-
Weekdays	Reference	Reference	Reference	Reference
Weekend	1.1	1.0	1.3	0.0366
Source of referral	-	-	-	-
Self-referral	Reference	Reference	Reference	Reference
Primary healthcare	0.1	0.0	0.6	0.0116
Ambulance	NA	NA	NA	NA
Other healthcare institution	0.7	0.2	2.5	0.6139

## Discussion

This retrospective study assessed the incidence, associated factors, and predictive factors of IVs to the ED in February 2023 at the National Guard Hospital, Makkah region, Saudi Arabia. The total number of visits was 5,429 and the incidence of IVs was 20.77%. Males and females contributed equally to the visits, and patients aged <10 years comprised almost one-fifth of the visits.

Our study identified several predictive factors of IVs in the ED, shedding light on the demographic and temporal patterns influencing healthcare-seeking behavior. First, male sex was found to be a significant predictor of IVs, with male patients more likely to present with non-urgent conditions in the ED. This finding aligns with the existing literature suggesting that males tend to utilize emergency services more frequently for non-emergent issues than females [6]. Understanding this gender disparity is crucial for targeted interventions aimed at promoting appropriate healthcare utilization among the male population.

Second, being non-Saudi was identified as another significant predictive factor for IVs in our study. Our study was conducted in a government hospital that mainly treats Saudi patients. Most non-Saudi patients treated in hospitals are hospital staff members or their relatives. This fact may reflect barriers to accessing PHC or cultural differences in healthcare-seeking behaviors. This might also reflect the fact that some of the IVs might have been caused by the staff themselves, necessitating further promotion of appropriate methods to seek medical attention among them. Similar findings have been reported in previous studies, highlighting the importance of addressing healthcare disparities among immigrant populations [10].

Additionally, visiting the ED on the weekend emerged as a predictive factor of IVs in our analysis. This temporal pattern has been observed in other studies. It may be attributed to limited access to PHC services or perceptions of convenience associated with weekend ED visits instead of working days. This further emphasizes the importance of ensuring access to PHC services outside of regular office hours [11].

Conversely, our study also identified negative predictive factors of IVs to the ED, indicating that demographic and referral patterns are associated with appropriate healthcare-seeking behavior. Notably, all age categories were significantly negative predictors of IVs when compared to the reference group of patients aged <10 years. Older age groups, particularly those aged ≥70 years, were less likely to present with non-urgent conditions in the ED. This finding underscores the importance of age as a determinant of healthcare utilization patterns, as older individuals exhibit more appropriate healthcare-seeking behaviors [12]. However, patients aged <10 years are usually brought to the ED by their parents or caregivers whose overly worried behavior could have resulted in them presenting to the ED unnecessarily. 

Furthermore, visits by patients referred from PHC settings were identified as negative predictors of IVs in our analysis. This indicates the effectiveness of PHC in diverting low-acuity cases from the ED. This highlights the critical role of PHC in triaging and managing patients with non-emergent healthcare needs, ultimately reducing unnecessary ED visits [13].

Our study also examined the association between presenting complaints and visit appropriateness, providing insights into the clinical context surrounding IVs. Visits with pulmonological and otorhinolaryngological complaints were significantly more likely to be inappropriate, suggesting the potential overutilization of ED resources for respiratory, ear, nose, and throat (ENT) conditions. Therefore, it is important to highlight these patient groups. In particular, almost one-fifth of the visits in our study presented with pulmonological complaints, and failure to adhere to the appropriate medical attention-seeking behaviors can heavily affect the ED. This finding underscores the need for targeted interventions to enhance access to alternative care settings for patients with such complaints, including PHC clinics and specialty outpatient services [14].

In contrast, visits with trauma/injury, cardiovascular, neurological, obstetric, gynecological, and genitourinary complaints were significantly more likely to be appropriate. These findings reflect the critical role of ED in managing acute and potentially life-threatening conditions across a range of medical specialties. Identifying these groups is crucial to provide appropriate management. Timely access to emergency care for patients with serious medical concerns is essential to optimize clinical outcomes and reduce the morbidity and mortality associated with these conditions. 

Overall, these observations contribute to the understanding of the presenting complaints that influence inappropriate ED visits and underscore the importance of targeted interventions to promote appropriate healthcare utilization and optimize resource allocation in the ED setting.

Several studies have evaluated the topic of IVs in different settings and using various criteria. A cross-sectional study was conducted in 2013 in all EDs in France to explore the socioeconomic and territorial factors associated with IVs in EDs [1]. Among the 29,407 patients in this study, 13.5% to 27.4% of ED visits were considered IVs. The likelihood of inappropriate use decreased in older patients but increased among young and female patients [1].

Another cross-sectional study on ED attendance was conducted in England and included 15,056,095 visits [6]. A total of 11.7% were categorized as IVs, with their rates being highest in those under 16 years of age and lowest in those over 85. In addition, being male and visiting on weekends had higher rates of IVs than the other counter groups [6].

The third retrospective study was conducted in Turkey and included 87,528 adult patients who visited the ED in 2017 [15]. They found that 9.9% of visits were non-emergent [15].

Another retrospective study was conducted at a tertiary hospital in Singapore in 2015 and included 120,606 visits [9]. They found that 9.6% of them were IVs. Moreover, the odds of IVs were higher among younger and self-referred attendees [9].

Finally, a systematic review published in 2009 aimed to assess the prevalence and associated factors of IVs, including 31 published articles [16]. The authors concluded that the prevalence of IVs in the literature ranges from 20% to 40%. Furthermore, they found that female patients, medically free patients, patients without a regular physician/source of care, patients, and those not referred to the ED by a physician have higher possibilities of having IVs [16].

Local studies on the topic of IVs in ED are mostly descriptive. One study conducted in the Al-Kharj Military Hospital in 1999 aimed to assess the inappropriate utilization of ED among 3,928 patients [7]. They found that 59.4% of patients had PHC or non-urgent problems. The most common complaint among the total was respiratory tract infections [7].

Several factors make it difficult to compare the findings of these articles with ours, including using different criteria to determine the IVs, using different statistical analysis tests, and reporting different variables. Despite these differences, the similarities can still be evaluated. Similar to our study, all studies reported high rates of IVs among their populations, ranging from 9.6% to 59.4% [1,6,9,15]. Moreover, several common findings were noted in both our findings and the literature to be associated with IVs, such as being male, self-referred, and young, which suggests their high impact. 

Patients’ motives for having IVs were vague in our study. One study revealed that patients with non-urgent conditions seek care in the ED because, even when they know their problem is not life-threatening, they fulfill their healthcare needs, with pain and anxiety being their main motivating reasons. In addition, patients who are well informed about the healthcare system and PHC services are more likely to choose the ED as a discerning health consumer. Ultimately, patient decisions are influenced by the convenience of being seen in the ED, access to technical facilities, and medication availability. These advantages save them from the complexities and organizational concerns that occur in other settings [17].

Another local study was conducted in Riyadh, Saudi Arabia, in which 350 people were surveyed to investigate why Saudi patients visit the ED for non-urgent conditions [8]. They found that not having a regular physician or a source of health care, the desire to receive care on the same day, and convenience for patients who prefer medical treatment that is available 24/7 were the main reasons why the patients attended the ED rather than the PHC centers [8]. Tackling and addressing the concerns of patients can be one of the first steps toward changing their behavior and understanding of ED services [18].

This study is not without limitations. The results of this study can be affected by the subjective nature of its retrospective design, as data collection via chart review is subject to missing information. In addition, including only a single tertiary center may not produce generalizable or epidemiological findings. Furthermore, both of the papers acknowledge that these criteria may not be error-free as it does not account for many factors such as medical history, educational background, socioeconomic status, mental health, and vital signs. Also, the choice of whether or not to order investigations lies completely on the covering ED physician opinion, which can introduce subjectivity in classifying the visits.

Although these limitations remain valid, this study contributes to the local evidence with a large sample size covering the topic of IVs in the ED in Saudi Arabia. Future research efforts should focus on prospective multicenter studies to validate our findings and explore additional factors influencing inappropriate ED utilization. In addition, we recommend developing a proper classification of IVs in EDs that considers the different potentially relevant factors we did not include in this paper. Lastly, we advocate establishing proper facilities that patients with non-urgent concerns can turn to for reassurance, guidance and needed medical treatment.

## Conclusions

In conclusion, our findings revealed a high incidence of inappropriate visits (IVs) in the Emergency Department. Being male, being non-Saudi, and visiting on the weekend were significantly positive predictors of the visit being inappropriate. Being older than 10 years and being referred from primary healthcare (PHC) were significantly negative predictors of IVs. Highlighting this topic is the first step toward minimizing the incidence of IVs and managing their related factors. It also emphasizes the need for targeted interventions aimed at promoting appropriate healthcare utilization, optimizing resource allocation, and improving the efficiency of emergency care delivery. This can improve not only the overcrowding in Eds but also the quality of healthcare delivered to patients in need and their clinical outcomes.
